# Speak My Language and I Will Remember Your Face Better: An ERP Study

**DOI:** 10.3389/fpsyg.2017.00709

**Published:** 2017-05-09

**Authors:** Cristina Baus, Jesús Bas, Marco Calabria, Albert Costa

**Affiliations:** ^1^Center for Brain and Cognition, Universitat Pompeu FabraBarcelona, Spain; ^2^Institució Catalana de Recerca i Estudis AvançatsBarcelona, Spain

**Keywords:** foreign language, social categorization, face recognition, old/new ERP effects, familiarity, recollection

## Abstract

Here we investigated how the language in which a person addresses us, native or foreign, influences subsequent face recognition. In an old/new paradigm, we explored the behavioral and electrophysiological activity associated with face recognition memory. Participants were first presented with faces accompanied by voices speaking either in their native (NL) or foreign language (FL). Faces were then presented in isolation and participants decided whether the face was presented before (old) or not (new). The results revealed that participants were more accurate at remembering faces previously paired with their native as opposed to their FL. At the event-related potential (ERP) level, we obtained evidence that faces in the NL were differently encoded from those in the FL condition, potentially due to differences in processing demands. During recognition, the frontal old/new effect was present (with a difference in latency) regardless of the language with which a face was associated, while the parietal old/new effect appeared only for faces associated with the native language. These results suggest that the language of our social interactions has an impact on the memory processes underlying the recognition of individuals.

## Introduction

In this globalized world, interaction with individuals in a language other than our mother-tongue is increasingly common. Consider for instance the following situation: a Spanish native speaker who is proficient in English is interviewing Spanish and English speaking candidates in their native language (NL) for a job. The interviewer will probably encounter more difficulties in understanding candidates in a foreign language (FL) (English) than in the NL (Spanish). But, will the language used during the interview influence the subsequent recognition of the candidates and therefore the final candidate selection? In other words, does the language in which we interact with others affect subsequent memory of those individuals? Here we explore whether and when memory processes underlying face recognition are affected by the language used by the individuals, native or FL.

There is evidence that language (or accent) context influences not only linguistic processing but also our social preferences. For instance, Kinzler et al. (2007) observed that infants show a preference for speakers using the infant’s NL. In one study, 5-months old English infants were presented with two women, one speaking their NL and the other a FL. Subsequently, when the two faces were presented silently, side-by-side, infants looked longer at the woman who previously spoke the infant’s NL (see also, Kinzler et al., 2009). In addition, adults tend to judge (in terms of intelligence or desirability) speakers of their NL more favorably than those speaking a FL (e.g., Lambert et al., 1960; Hansen et al., 2014) and speakers with a native accent sound more credible than those with a foreign accent (Lev-Ari and Keysar, 2010). Thus, interactions involving a FL (or a foreign accent) are not just more difficult linguistically, but also appear to be treated differently at the social level. In the present study, we go one step beyond and characterize for the first time the impact of language context on the processing of an individuals’ face.

There are several reasons why one may expect an effect of language on the processing of attributes of other individuals. The first one relates to social categorization. For instance, Pietraszewski and Schwartz (2014) recently showed that when we confuse one person identity, it is more likely that we do it with a person of the same accent than with a person of the different accent. This suggests that accent may be an important dimension of social categorization (see also Rakić et al., 2011; Hansen et al., 2017). These studies, however, do not directly test whether language context actually affects how well faces are recognized.

Importantly here, there is ample evidence suggesting that when individuals are categorized as out-group members, their corresponding faces are less well recognized, as shown by the “other-gender effect” (e.g., Wolff et al., 2014) the “other-age effect” (e.g., Wiese, 2012) or the well documented “other-race effect” (e.g., Hugenberg et al., 2010).^1^ More crucially for present proposes, social categorization influences face recognition even when the properties of the faces are held constant (e.g., faces of the same race). Bernstein et al. (2007) showed that labeling a face as out-group automatically hindered its recognition (see also, Hehman et al., 2011; Ng et al., 2016). For example, participants are less accurate at recognizing those faces that have been previously associated with another university (out-group member) than those associated with their own university (in-group member) (see also Shriver et al., 2008; Rule et al., 2010; Herzmann and Curran, 2013, for in-group/out-group distinctions based on personality type, socioeconomic status or religious affiliation). Thus, just believing that a target is a meaningful in-group member is enough to increase attention during facial encoding, which in turn leads to better recognition. In this context, it seems inevitable to think that language, as a powerful source of social categorization, will modulate memory processes underlying face recognition as belonging to the same University does.

The second reason to expect an effect of language on face recognition relates to the processing burden faced by comprehenders when listening to a FL. This is often revealed by slower response times and more errors when processing a FL than a NL (e.g., Hahne, 2001). Importantly here, it is necessary to assume that such “processing cost” will have consequences for memory recognition not only for verbal information, but for visual information as well (i.e., cross-modal interference). For accounts such as the multiple resources model in multitasking (Wickens, 2008), the effect of language on face recognition would stem from the more resources needed to process sentences in a FL than in a NL. In particular, the extra resources allocated for processing a FL would result in fewer resources available for encoding the face, thus hampering its encoding and later recognition. The most direct test of cross-modal interference comes from more applied psychological domains, such as eyewitnesses’ memory (e.g., Staller, 2010; Pickel and Staller, 2012). For instance, in Pickel and Staller’s (2012) study, participants were presented with a scene in which they were asked to imagine themselves as the manager during a bank robbery. Bank-robbers could speak either with a native or a foreign accent. When participants were later asked they retrieved more details about a bank-robber when speaking with a native accent than with a foreign accent. Nevertheless, when participants were asked to recognize the bank-robber in a photo line-up, no accent effect was observed. These results suggest that differences of cognitive processing in one modality (e.g., language/accent) do not interfere with recognition of all aspects on the other modality (e.g., visual properties; see also Ellis, 1975, for race not affecting voice recognition). This posits some limits to models assuming cross-modal interference (e.g., Wickens, 2008).

In sum, while social and cognitive accounts would predict an impact of the language spoken on the subsequent recognition of a face, the experimental evidence is very scarce and rather elusive. On the one hand, there is evidence of social categorization by language (e.g., accent; Pietraszewski and Schwartz, 2014), but not whether this determines the success in the recognition of a face. On the other hand, while processing of a FL requires more cognitive resources than processing of a NL (Pickel and Staller, 2012), this does not seem to affect the subsequent recognition of a face. Thus, the novelty of the present study lies in testing how the language context determines how accurate a face will be recognized. Therefore, the present study is very relevant for two reasons: (1) at the practical level, it allows describing how FL influences our social interactions, an important phenomenon nowadays, especially since English has become more and more present in several social contexts, and (2) at the theoretical level, it has the potential to extend our knowledge about the influence of non-visual information such as language in modulating face recognition.

To investigate the influence of language on face recognition, here we adapted the old/new paradigm to the context of language. The old/new paradigm has been extensively used in the literature on memory (e.g., Rugg et al., 1998; Bernstein et al., 2007) and it is therefore a very suitable paradigm for exploring the effects of language context on face recognition (e.g., Allan et al., 1998; Curran, 2000; Finnigan et al., 2002; Paller et al., 2003; see Friedman and Johnson, 2000; Rugg and Curran, 2007, for reviews). In our task, participants had to decide in a recognition phase whether a given face had been presented (old) or not during the previous encoding phase. In the recognition phase, the faces were presented without any acoustical information. However, during the encoding phase (previous to the recognition phase) half of the faces were presented along with sentences in the participants’ NL, and the other half with their NL. In addition, we recorded electrophysiological activity while participants made old/new judgments.

At the electrophysiological level, the retrieval of information from memory has been associated with the “ERP old/new effect,” calculated as the difference between event-related potentials (ERPs) elicited by those trials correctly recognized as old and those correctly recognized as new. Importantly here, ERP old/new effects appear to be sensitive to the categorization of a face as in-group or out-group: larger old/new effects have been reported for in-group faces regarding race (Stahl et al., 2010; Herzmann et al., 2011; Derks et al., 2014; Wiese et al., 2014), age (Wiese et al., 2008; Wiese, 2012), gender (Wolff et al., 2014) or personality type (Herzmann and Curran, 2013). Additionally, the in-group memory advantage has been mainly associated with two ERP components, the frontal (e.g., FN400, 300–500 ms after target presentation; e.g., Curran and Hancock, 2007) and the parietal old/new effects (LPC, 500–700 ms; Herzmann et al., 2011; Herzmann and Curran, 2013). Differences between in-group and out-group faces appear to be especially evident at parietal sites, taken as an indication that more details are retrieved during in-group face recognition. In a similar vein, the frontal and parietal old/new effects are also sensitive to the depth to which stimuli are encoded (e.g., Rugg et al., 1998; Marzi and Viggiano, 2010). While stimuli encoded shallowly (few cognitive resources devoted) elicit the frontal old/new effect, stimuli deeply encoded (more cognitive resources devoted) elicited both the frontal (FN400) and the parietal (LPC) old/new effects. Altogether, these results provide clear hypotheses for our study: If faces paired with the NL are categorized as in-group or more cognitive resources are devoted to their encoding (as fewer resources are devoted to the processing of the NL), then faces paired with the NL should lead to a better recognition than faces paired with the FL. In addition, better recognition memory (larger old/new effect) is expected to be indexed by a more sustained activity at parietal sites.

In addition to the old/new effects, we explored potential differences between ERPs associated with language context in the encoding phase. This is important because while the old/new effect has been interpreted as reflecting the way in which faces are encoded to our knowledge no study has directly explored this issue in the context of language and face recognition^2^.

In sum, our aim was to explore the impact of language processing on memory for faces. We did so by assessing whether face recognition is affected by the language (native vs. foreign) associated with the faces during the encoding phase.

## Materials and Methods

### Participants

Forty-two Caucasian Spanish native speakers from the University Pompeu Fabra (Barcelona) took part in this experiment (mean age = 21, *SD* = 2.07, 18 women) in exchange for monetary compensation. They all declared not having any visual, hearing, nor neurological problems and English was their FL (see **Table 1**). Speech comprehension in the FL was evaluated by asking participants to listen to a 6 min recording and then respond to different eight comprehension questions. On average, participants responded correctly to 6.3 questions (*SD* = 1.3) indicating a good level of English proficiency.

**Table 1:** Self-reported proficiency (ranging from 1 = very low to 10 = native-like proficiency), percentage of daily use and average score in the comprehension test.

Language proficiency	NL-Spanish	FL-English
Comprehension	9.96 (0.3)	7.4 (1.1)
Reading	9.96 (0.17)	7.9 (1.2)
Speaking	9.96 (0.17)	6.9 (1.4)
Pronunciation	9.96 (0.17)	6.9 (1.5)
Writing	9.90 (0.3)	7.5 (1.1)
% Daily use	79.8 % (12)	20.2% (1.2)
FL comprehension test (0–10)	–	7.66 (1.9)

From the initial pool of participants, one was discarded because of problems during the recording session. Moreover, as will be described in the ERP recording section, eight participants were excluded for different reasons. Thus, the final pool included 33 participants (mean age = 20.9; *SD* = 2; 16 women).

### Materials

Eighty gray-scale photographs of Caucasian faces (half male and half female) were downloaded from free electronic datasets and other resources on the web. All of them were emotionally neutral and had no extra visual details (e.g., earrings).

Forty non-autobiographical sentences were created and then recorded in Spanish and English (“*El ordenador es muy caro*,” “*The computer is very expensive*”). Sentences ranged from 4 to 7 words in length in Spanish (mean = 5.2 words) and in English (mean = 4.9 words). Twenty native Spanish speakers (10 males and 10 females) and 20 English native speakers (10 males and 10 females) recorded the sentences. Therefore, a given sentence could be produced by four speakers: Spanish female, Spanish male, English female, and English male. Recording durations for sentences in Spanish and English (considering the female and male voice for each sentence) did not differ significantly [1,464 s vs. 1,452 s, *t*(79) = 0.309, *p* = 0.75]. Thus, the final design consisted of photographs of faces accompanied by a voice speaking either in Spanish (NL) or in English (FL). Across participants, faces were presented in all conditions (Spanish old, English old, and new conditions) and sentences were associated with Spanish or English faces, females and males. That is, faces and sentences were cycled through the different conditions across participants.

To ensure that sentences were understandable for the participants, a new group of participants (*n* = 13) were asked to translate the English sentences into Spanish. English proficiency of the new pool of participants was similar to that indicated by the participants in the experiment (comprehension: 7.6, reading: 7.6, speaking: 6.8, pronunciation: 6 and writing: 7). After translation, the sentences were coded with a 1, 0.5, or 0 depending of whether the sentence was correct, correct except for one word, or incorrect, respectively. The results showed that participants correctly translated 85% of the 40 sentences (*SD* = 5.4), indicating that sentences were clearly understandable for the participants.

### Procedure

The experiment consisted of two phases: the encoding and the recognition phases. In the encoding phase, face photographs were displayed along with the auditory presentation of the sentences (SOA 0). Participants were instructed to pay attention to the faces and the sentences, because later they would have to do a task related to the encoding phase, but without explicitly mentioning that it would be a recognition task. The trial structure was as follows: an asterisk was presented on the screen for 500 ms followed by the simultaneous presentation of the face and the auditory sentence (either in Spanish or in English) for 2000 ms. Upon completion of the encoding phase, participants engaged in a distractor filler task for 5 min (Tetris game) to avoid having the recognition phase immediately after the encoding phase.

After that, participants started the recognition phase, in which photographs were presented in silence. Participants were instructed to identify by means of two keys on a keyboard whether a given face was old or new. The assigned key for new and old were counterbalanced across participants. A given trial included: an asterisk appearing for 500 ms followed by the presentation of the face, which remained on the screen until the participant’s response. Eighty faces were presented in this phase, 40 that were already presented in the encoding phase (20 in Spanish and 20 in English) and 40 new faces.

Finally, we asked participants to perform the same old/new judgment for the sentences. To do so, old sentences (*n* = 40) and new sentences (*n* = 40) were visually presented to the participants to avoid any confound with voice identification. Half of the sentences were in Spanish and half in English. A trial was comprised of the presentation of an asterisk (500 ms) followed by the presentation of a sentence in the center of the screen that remained until participants judged whether the sentence was old or new. The results from this task allowed us to ensure that participants were paying attention to the sentences during the encoding phase.

### Data Coding

Correct and incorrect responses were coded during the recognition phase. Depending on the response of the participant and the type of trial, four types of responses were coded: Hits (old trial/correct response), misses (old trial/incorrect response), correct rejections (Crej, new trial/correct response) and false alarms (new trial/incorrect response), with misses and false alarms being complementary to Hits and Correct Rejections, respectively. In the face recognition task, given that “new” faces were not separated for language, instead of calculating the *d*′ as a measure of sensitivity, accuracy analyses were based on the comparison between the three types of trials: hits for faces previously paired with the NL, hits for faces paired with the FL and correct rejections for new faces. For the sentence recognition task, since both “old” and “new” were separated by language, we calculated the *d* prime [*d*′ = Z (Hits) - Z (False Alarms)] as a measure of discrimination sensitivity.

### ERP Recording and Analysis

The EEG was continuously recorded from 60 Ag-AgCl electrodes, mounted on an elastic cap (ActiCap, Munich, Germany) and positioned according to the international 10–20 system. Moreover, three external electrodes (EOG) were placed above, below and on the outer canthus of the right eye to register vertical and horizontal eye movements, respectively. All active electrodes were on-line referenced to the left mastoid. Impedances were kept below 15 kΩ. EEG data was sampled at 500 Hz with a bandpass of the hardware filter of 0.1–125 Hz. Data was filtered offline (0.1–30 Hz) and re-referenced to the average activity of the two mastoids. An Independent Component analysis (ICA) was employed to detect and correct eye blink artifacts (SemiAutomatic FastICA decomposition, 25 components). Only those components clearly indexing vertical and horizontal eye-movements were selected and corrected. EEG was then epoched from 200 ms before to 1000 ms after stimulus onset. Epochs with amplitudes above or below +100 μV or with a difference between the maximum and the minimum amplitude larger than 75 μV were considered artifacts and discarded from the analysis. Participants without a sufficient number of trials (less than 10 trials per condition) due to excessive ocular artifacts (*n* = 4), low trial counts (less than 9 trials/condition, *n* = 3) or an excessive number of bad channels (*n* = 1) were discarded. Thus, the final analysis included 33 participants. Averages were calculated for the encoding and the recognition phases separately. In the encoding phase^2^, averages for faces in the NL condition (Encoding NL) and in the FL (Encoding FL) were compared (20 trials per condition). In the recognition phase, averages were calculated for three types of trials: Hit-NL (correct responses to old faces that were paired with the NL; average number of epochs across participants = 13.5, *SD* = 2.1), Hit-FL (correct responses to old faces paired with the FL; 11.9 epochs, *SD* = 2.1) and Correct rejections (correct responses to new faces; 28 epochs, *SD* = 3.6). The number of trials was statistically different across the three conditions (all *p*’s < 0.01).

For the ERP analyses in the encoding phase, we selected an early time-window around the P200 (150–230 ms) and a late time-window around the Late Positive Component -LPC-, component (600–800 ms), two ERP components previously related to the detection of acoustic differences/social categorization and to memory processes, respectively (e.g., Wiese et al., 2008; Romero-Rivas et al., 2015). Selection of the P200 time-range was made by selecting the maximal peak across electrodes (around 190 ms) and including 40 ms before and after it (150–230 ms). A similar procedure was followed for the LPC time-window, with the exception that the time range comprised 100 ms before and after the maximal peak (around 700 ms).

We considered the factors: type of trial (Encoding-NL, Encoding-FL), region (frontal/parietal) and laterality (left, right), leading to four regions of interest (ROIs): Left frontal (Fp1, AF3, F1, F3: LF), Right frontal (Fp2, AF4, F2, F4: RF) Left parietal (P3, P1, CP3, CP1: LP), and Right parietal (P4, P2, CP4, CP2: RP).

For the ERP analyses in the recognition phase, to identify time-windows of interest, we combined a priori knowledge based on previous literature with an assumption free procedure, the cluster-based permutation test. In a first analysis (hereafter main analysis), time-segments and ROI were defined according to previous research considering early and late old/new effects during face recognition (e.g., Yovel and Paller, 2004; MacKenzie and Donaldson, 2007; Yick and Wilding, 2014). In particular, early old/new effects were explored between 300 and 500 ms and late old/new effects between 500 and 700 ms. Mean amplitudes at each time-window of interest were computed by averaging the activity of electrodes within each region, condition and participant. We considered the factors: type of trial (Hit-NL, Hit-FL, Crej), region (frontal/parietal) and laterality (left, right), leading to four ROIs: Left frontal (Fp1, AF3, F1, F3: LF), Right frontal (Fp2, AF4, F2, F4: RF), Left parietal (P3, P1, CP3, CP1: LP), and Right parietal (P4, P2, CP4, CP2: RP). Secondly, although time-windows and ROIs were selected on the basis of previous studies and therefore independently of differences between our conditions, to further estimate reliable amplitude differences (Type I errors; Kilner, 2013) we conducted a paired two-tailed cluster-based permutation test (i.e., maximum cluster-level mass; Groppe et al., 2011). The test does not make any *a priori* assumption on when and where an effect might occur, thus limiting the possible confounding issues due to multiple comparisons. Based on the cluster-permutation test, old/new effects were further explored in a more focused time-range (464–590 ms) and electrodes (frontal-central; see **Figure 4**).

Significance levels of the *F* ratios were adjusted with the Greenhouse–Geisser correction. Where the ANOVA revealed a significant effect of type of trial, old/new effects (comparing Hits vs. Crej) were further evaluated for each language separately with paired-samples *t*-tests. Effects sizes are reported as partial eta-squared ($\eta_{p}^{2}$) or Cohen’s *d* (*d*) values.

## Results

### Behavioral Results

#### Recognition Memory for Faces

**FIGURE 1:**
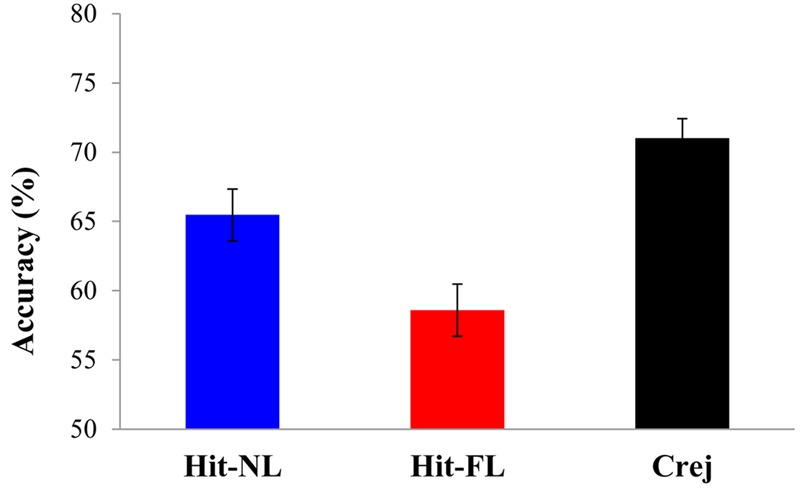
**Mean accuracy in the recognition memory for faces for hit-NL (blue bar), hit-FL (red bar) and correct rejections (black bar) Error Bars depict mean standard errors**.

Recognition accuracy was different from chance for Hits in the NL [65.9%; *t*(32) = 8.05, *p* = 0.000, *d* = 1.4] and in the FL conditions [58.7%; *t*(32) = 4.16, *p* = 0.000, *d* = 0.7] and for correct rejections [71.3%; *t*(32) = 13, *p* = 0.000, *d* = 2.2]. Recognition accuracy for faces was further explored in a one-way ANOVA comparing the three types of trials: old faces paired with the NL (Hit-NL), old faces paired with the FL (Hit-FL) and new faces (Crej). The results revealed a main effect of type of trial [*F*(2, 64) = 10.7, MSE = 121.7, *p* = 0.000, $\eta_{p}^{2}$ = 0.38] (see **Figure 1**). Pairwise comparisons revealed that participants were more accurate recognizing new than old faces [Crej vs. Hit-NL: *t*(32) = 2.05, *p* = 0.04, *d* = 0.35; Crej vs. Hit-FL: *t*(32) = 4.4, *p* = 0.000, *d* = 0.76]. Importantly, language had an impact on face recognition: faces paired with the NL were recognized more accurately than faces paired with the FL [*t*(32) = 2.6, *p* = 0.01, *d* = 0.46].

#### Recognition Memory for Sentences

Sentence recognition served mainly to assess whether participants were attending to the sentences during the encoding phase. Three participants were not included in these analyses because of a technical problem during this phase. Thus, analyses included 30 participants. The results showed that accuracy for hits [NL: 58%; *t*(29) = 2.7, *p* = 0.009, *d* = 0.5; FL: 54%, *t*(29) = 1.4, *p* = 0.14] and correct rejections differed from chance [NL: 79%; *t*(29) = 10.4, *p* = 0.000, *d* = 1.9; FL: 72%; *t*(29) = 7.7, *p* = 0.000]. The results considering the discrimination accuracy (*d*′) for sentences in Spanish (*d*′ = 1.17) and English (*d*′ = 0.87) revealed an advantage when identifying sentences in the native relative to those in the FL [*t*(29) = 2.00, *p* = 0.05, *d* = 0.36].

### Electrophysiological Results

#### Encoding Phase

**FIGURE 2:**
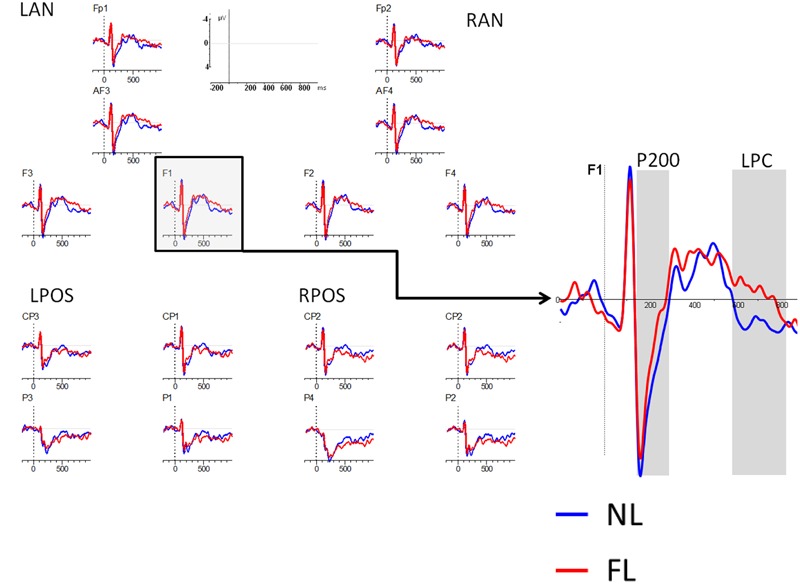
**ERPs during the encoding phase.** Lines represent encoding trials in the NL condition (blue line) and encoding trials in the FL condition (red line). Negative is plotted up. Right figure represents a zoomed figure of the electrode F1.

As mentioned previously, analyses in the encoding phase included the factors: type of trial (NL, FL), region (frontal/parietal), and laterality (left, right), leading to four ROIs: Left frontal (Fp1, AF3, F1, F3: LF), Right frontal (Fp2, AF4, F2, F4: RF), Left parietal (P3, P1, CP3, CP1: LP), and Right parietal (P4, P2, CP4, CP2: RP). Two time-windows were explored: one early, the P200 (150–230 ms), and one late, the LPC component (600–800 ms) (see **Figure 2**).

##### 150–230 ms

The results revealed a main effect of type of trial [*F*(1,32) = 5.01, MSE = 1.95, *p* = 0.03, $\eta_{p}^{2}$ = 0.13] with NL encoding eliciting more positive amplitudes than FL encoding. The effect was larger for those electrodes left-lateralized than for those on the right side of the scalp as indicated by the interaction between type of trial and laterality [*F*(1,32) = 4.17, MSE = 0.32, *p* = 0.049, $\eta_{p}^{2}$ = 0.11]. No other interaction with type of trial showed significance, revealing that differences between conditions were present across ROIs.

##### 600–800 ms

The effect of type of trial was not significant (*F* < 1), but it interacted significantly with region [*F*(1,32) = 5.3, MSE = 17.4, *p* = 0.016, $\eta_{p}^{2}$ = 0.14], revealing a larger positivity for ERPs in the NL than in the FL in the frontal regions [*F*(1,32) = 4.1, *p* = 0.04, $\eta_{p}^{2}$ = 0.12] but not in the posterior ones (*F* < 1).

The results in the encoding phase revealed that both for the P200 (150–230 ms) and the LPC (600–800 ms) components, encoding of faces presented with the NL elicited larger amplitudes than encoding of faces presented with the FL. While early differences can be interpreted in terms of acoustic/categorization differences, late components are more directly related to memory encoding. We will take these results back in the “Discussion” section.

#### Recognition Phase: Main Analyses

**FIGURE 3:**
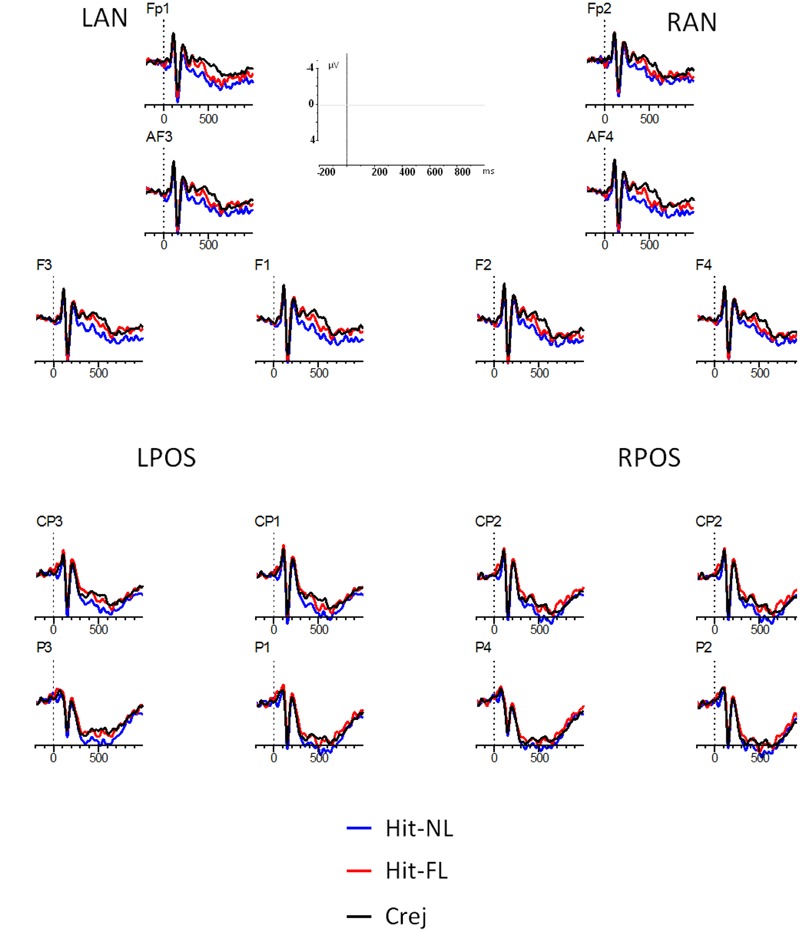
**ERPs during the face recognition task.** Lines represent Hits in the NL condition (Hit-NL, blue line), Hits in the FL condition (Hit-FL, red line) and correct rejections (Crej, black line). Plotted electrodes represent the four regions of interest (ROI): Left frontal (Fp1, AF3, F1, F3: LF), Right frontal (Fp2, AF4, F2, F4: RF), Left parietal (P3, P1, CP3, CP1: LP), and Right parietal (P4, P2, CP4, CP2: RP). Negative is plotted up.

The main analyses included the factors: type of trial (Hit-NL, Hit-FL, Crej), region (frontal/parietal), and laterality (left, right), leading to four ROIs: Left frontal (Fp1, AF3, F1, F3: LF), Right frontal (Fp2, AF4, F2, F4: RF), Left parietal (P3, P1, CP3, CP1: LP), and Right parietal (P4, P2, CP4, CP2: RP). **Figure 3** represents the grand averages for each condition (Hit-NL, Hit-FL, Crej) at the four selected ROIs.

*300–500 ms.* The results in the time-window between 300 and 500 ms revealed a main effect of type of trial [*F*(1.9,63.5) = 5, MSE = 71.1, *p* = 0.01, $\eta_{p}^{2}$ = 0.13]. Pairwise comparisons revealed that Crej differed significantly from Hit-NL [*t*(32) = -3.04, *p* = 0.005, *d* = 0.5] but not from Hit-FL (*t* < 1) suggesting an old/new effect for those faces associated with the NL but not for those associated with the FL. This result was further validated by a significant difference between Hit-NL and Hit-FL [*t*(32) = 2.4, *p* = 0.02, *d* = 0.4]. Type of trial did not interact with region (*F* < 1) or laterality [*F*(1.8,58) = 2.8, MSE = 18.5, *p* = 0.07, $\eta_{p}^{2}$ = 0.08], suggesting that old/new effects for those faces paired with the NL were fronto/parietally distributed (see **Figure 3**).

*500–700 ms.* A main effect of type of trial [*F*(1.7,57) = 4.01, *p* = 0.02, MSE = 79.7, $\eta_{p}^{2}$ = 0.11] revealed that Crej differed significantly from Hit-NL [*t*(32) = -3.1, *p* = 0.004, *d* = 0.5] but not from Hit-FL [*t*(32) = -1.2, *p* = 0.2, *d* = 0.2]. However, Hit-FL did not differ from Hit-NL [*t*(32) = 1.4, *p* = 0.16, *d* = 0.2], indicating that both conditions did not differed from each other. None of the other interactions involving type of trial resulted significant (all *F*s < 1) (see **Figure 3**).

The main analysis revealed early differences in the recognition of faces depending on the language to which they were previously paired. Between 300 and 500 ms old/new effects at frontal and parietal regions were modulated by language, being only present for those faces paired with the NL of the participants.

#### Recognition Phase: Focused Analyses

**FIGURE 4:**
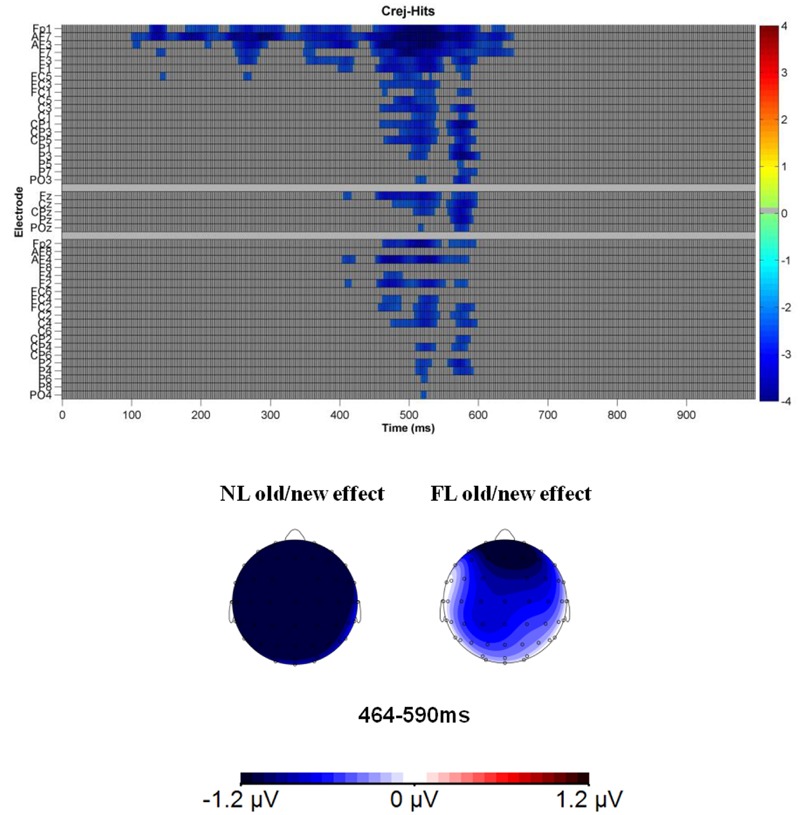
**Raster diagram illustrating significant differences between ERPs to Crej and Hits (regardless of language) in the face recognition phase according to a *cluster* permutation test.** Blue rectangles indicate electrodes/time points in which the ERPs to Crej are more negative than Hits. Gray rectangles indicate electrodes/time points at which no significant differences were found. Note that electrodes are organized according to laterality and region. Left and right electrodes are grouped on the figure’s top and bottom, respectively. Midline electrodes are shown in the middle. Moreover, each group of electrodes is ordered from frontal to posterior electrodes. Lower figure represents the topographical map of the old/new effect in the time-window between 464 and 590 ms for NL and FL.

As mentioned previously, a paired two-tailed cluster mass permutation test was conducted (Bullmore et al., 1999; ERP toolbox, Groppe et al., 2011) in the time-range between 0 and 1000 ms and for 43 electrodes (excluding occipital and temporal electrodes). The threshold for cluster inclusion had an alpha-level of 0.03 and electrodes within approximately 4.1 cm of distance were considered spatial neighbors (average = 3.3; assuming a 56-cm head average head circumference). We computed 1000 permutations to estimate the distribution of the null hypothesis. This test revealed a negative cluster, showing that Crej were more negative than Hits in the range, maximal between 464 and 590 ms at 20 electrode sites (reaching 30 electrodes for some datapoints; see **Figure 4** for the raster graph with the significant *t*-test scores). As can be appreciated in the raster plot, in this latency range, the difference was more pronounced over frontal-central electrodes. In order to know the contribution of each language (NL and FL) to the observed old/new effect, mean amplitudes (464–590 ms) were explored by considering the factors: Type of trial (Hit-NL, Hit-FL, Crej), region (frontal and central) and laterality (left, right).

In the time window of interest (464–590 ms), the results revealed a significant difference between the trials [*F*(1.8,60) = 7.3, MSE = 20.8, *p* = 0.002, $\eta_{p}^{2}$ = 0.18]. Pairwise comparisons of the old/new effect (Hits vs. Crej) for each language revealed that the old/new effect (correct rejections eliciting more negative amplitudes than hits), was present for faces previously paired with the NL of the participants [2 μV; *t*(32) = -4.2, *p* = 0.000, *d* = 0.7] and also for those paired with their FL [1 μV; *t*(32) = -2.1, *p* = 0.03, *d* = 0.3]. In addition, no differences were obtained between Hits in the NL and FL, revealing similar old/new effects when recognizing faces previously paired with the NL than the FL of the participants [0.91 μV; *t*(32) = 1.5, *p* = 0.14, *d* = 0.2]. Type of trial did not interact with region or laterality (all *F*s < 1), revealing that old/new effects were fronto-centrally distributed.

These results show that the FN400 old/new effect was present for both languages, being the effect larger for those faces paired with the NL than with the FL of the participants.

Taken together, the ERP results revealed that the language to which a given face is paired has an impact during its recognition. Early frontal and parietal old/new effects (300–500 ms) were observed for those faces associated with the NL of the participants. In contrast, old/new effects appeared with a delay for those faces associated with the FL and restricted to anterior electrodes.

#### Internal Consistency of the Old/New Effect

One concern of our study is the small number of ERP trials considered in the analyses (Hit-NL: 13.5 epochs; Hit-FL: 11.9 epochs; Correct rejections: 28 epochs). To test the consistency of the reported effect, we computed the Cronbach’s alpha of the FN400 component^3^ (restricted analysis; 464–590 ms) averaged over 5, 10, and 13 trials^4^ for each condition at the electrode Fz (as the representative electrode). For the FN400 in the correct rejections condition, Cronbach’s alpha was calculated also for 20 and 25 trials. To determine the internal reliability, we followed Hinton’ (2004) classification: Cronbach’s alpha exceeding 0.90 indicates excellent internal reliability, between 0.70 and 0.90 indicates high internal reliability, from 0.50 to 0.70 indicates moderate internal reliability, and below 0.50 is low. The results showed that internal reliability of the FN400 amplitude increases as more trials are considered, with Cronbach’s alpha revealing a already a moderate reliability for 5 trials in all conditions (Crej α = 0.56, Hit-NL α = 0.61, Hit-FL α = 0.56) and 10 trials (Crej α = 0.66, Hit-NL α = 0.61, Hit-FL α = 0.76) reaching a high consistency when 13 trials were considered (Crej α = 0.70, Hit-NL α = 0.76, Hit-FL α = 0.81). The internal reliability kept high when 20 and 25 trials were considered for Crej (20 trials α = 0.77; 25 trials: 0.82). This is important in showing that despite the number of trials per condition was low, the FN400 component is stable (see similar consistency after few trials for the FRN component: Marco-Pallares et al., 2011; for the N200 component: Rietdijk et al., 2014).

## Discussion

The aim of this experiment was to investigate how language context, native or foreign, influences subsequent face recognition. To do so, we explored the behavioral performance and neural activity associated with recognition memory and how the underlying processes were modulated by language. In particular, we tested the hypothesis that faces paired with a NL would be encoded and consequently recognized more accurately than those paired with a FL. Indeed, this hypothesis turned out to be supported by the results since native speakers of Spanish were more accurate at recognizing faces that were previously paired with their own language than with a FL. At the ERP level, face encoding was associated with larger positivities, at the P200 and LPC, for faces paired with the NL than for faces paired with the FL. Face recognition was associated with the early frontal-parietal old/new effect (300–500 ms) for those faces paired with the NL. Instead, old/new effects for faces associated with the FL appeared with a delay and were restricted to the anterior region (fronto-central electrodes). That is, while frontal old/new effects were present regardless of the language with which a face was paired, parietal old/new effects were present only for faces paired with the NL.

Our results provide evidence that the language in which we interact with others has an impact on the processing of a face. Whether, this effect originates from social or cognitive factors cannot be distinguished with the present data. Faces presented with the FL might be socially categorized as out-group, leading to a less detailed encoding and consequently to a poorer recognition (relative to those associated with the NL). These findings are in line with social-categorization models (Sporer, 2001, for a review) and suggest that language, like the race or the University affiliation, is an important cue for social categorization (Kinzler et al., 2007; Pietraszewski and Schwartz, 2014) that bias our attention and memory toward those linguistically more similar to us (native speakers). Similarly, these findings are also in line with the idea of a “processing cost” in understanding sentences in a FL affecting encoding and subsequent recognition of a face. The greater cost entailed in processing a FL might result in fewer cognitive resources to process the face in detail, thus hindering its later recognition (e.g., Wickens, 2008; Pickel and Staller, 2012). Likewise, the own-language memory advantage might result from same-language faces receiving more cognitive resources (as fewer resources are dedicated to understand the language) than other-language faces during encoding (e.g., Herzmann and Curran, 2013).

While our results support both social and cognitive proposals, they contrast with previous evidence on eyewitnesses’ memory showing no advantage in recognizing perpetrators of the same accent (Staller, 2010). We do not have a ready explanation for the observed differences, but it is likely that methodological aspects such as, the tasks employed or how participants were instructed, might play a role. For instance, participants in Staller’s (2010) study were asked to pay attention to the message of perpetrators, which might have impaired attentional resources to process the face in detail. It is also possible that the old/new paradigm is a more sensitive paradigm to capture categorical/cognitive differences during face recognition. Note however, that although our results clearly replicate previous studies on the effects of social and cognitive factors on face recognition (e.g., Bernstein et al., 2007), their generalization is limited by the fact that we did not test a group of English listeners with the same materials, therefore providing a full-cross over interaction between language of the listener and memory recognition. Further research will help to determine the necessary conditions and the generalization the other-language effects in face recognition.

Our electrophysiological results also support the other-language effect during memory encoding and recognition. First, during the encoding phase, faces paired with the NL elicited enhanced P200 and LPC amplitudes relative to faces paired with the FL. Second, during the recognition phase, language did not modulate the frontal and parietal old/new effects to the same extent. ERPs elicited by faces previously paired with the NL showed larger effects and occurred across a broader time range and scalp topography than ERPs elicited by FL faces. These results provide insights into the memory mechanisms responsive for the superior memory encoding and recognition for faces in the NL condition.

Regarding the encoding phase, the enhanced P200 and LPC for faces presented with the NL (own-language faces) replicate previous studies on social categorization showing larger positivites for own-age faces (e.g., Wiese et al., 2008) and own-race faces (Stahl et al., 2008).^5^ These results have been interpreted as reflecting larger early perceptual and attentional processes (P200) as well as more elaborative memory processes (LPC) during episodic memory encoding. In this context, the NL would have received more attention and deeper processing than the FL, leading in turn to a more detailed encoding and to a better later recognition. However, given that faces and language were presented together in the encoding phase, the observed P200 differences might as well indicate differences in the identification/extraction of acoustic features during comprehension of the NL and the FL (Romero-Rivas et al., 2015).

Regarding the recognition phase, the old/new effects revealed an advantage for those faces previously paired with the NL. The observed parietal old/new effect for those faces paired with the NL clearly replicates previous findings showing an in-group memory advantage during face recognition regarding race, age, gender or personality type (e.g., Wiese, et al., 2008; Herzmann et al., 2011; Herzmann and Curran, 2013; Wolff et al., 2014). Additionally, while old/new effects for out-group faces have been more elusive, some studies have reported a link between out-group recognition and frontal old/new effects (e.g., Li et al., 2004; Stahl et al., 2008; Herzmann and Curran, 2011, 2013).

The frontal and parietal old/new effects have been mainly interpreted within the realm of dual-memory processes (Curran, 2000; for reviews see, Yonelinas, 2002; Yonelinas et al., 2010), as reflecting two distinct memory processes. Notably, frontal old/new effects (i.e., FN400) are interpreted as an index of *familiarity-based* recognition processes (i.e., unsubstantiated sense of previously encountering an item) and parietal old/new effects (i.e., LPC) as revealing *recollection processes*, which entail the recovery of information about the context or details about the encountered item (but see, MacKenzie and Donaldson, 2007). Within this framework, memory recognition of all faces, regardless of their language, would engage familiarity processes while only in-group faces would engage recollection processes. However, given that we did not ask participants to recall any information, our results might be revealing the contribution of frontal and parietal neural generators to familiarity processes during face recognition (Yovel and Paller, 2004; MacKenzie and Donaldson, 2007, 2009; see also Donaldson and Curran, 2007). In this context, the smaller, shorter, and more topographically restricted old/new effects for faces associated with the FL than for faces associated with NL might suggest differences in the strength to which familiarity processes are involved (Finnigan et al., 2002), instead of different memory processes. That is, faces in the FL sound less familiar to the participants as a consequence of weaker memory traces during encoding and retrieval. While further research is needed to differentiate between theoretical explanations regarding the frontal and parietal effects, here we provide evidence regarding the effects of language context, native or foreign, on the recognition of faces: faces associated with a FL are subsequently recognized more poorly than those associated with a NL. The ERP results suggest that differences between the NL and the FL start already at the encoding phase, affecting the strength of memory traces and the subsequent face recognition.

## Ethics Statement

Clinical Research Ethics Committee Report (Parc de Salut MAR). Participants were given a consent form before the experiment with information regarding the ERP procedure, clearly indicating the pros and cons of the ERPs technique for the comfort of the participant. Only when understood and signed by the participants, the experiment started. Participants were adults without neurological problems.

## Author Contributions

CB, JB, and AC designated the experiment. CB and JB conducted the experiment and analyzed the data. CB, JB, MC, and AC discussed the results. CB and JB prepared a draft and MC and AC critically reviewed it. All authors gave final approval of the version to be submitted.

## Conflict of Interest Statement

The authors declare that the research was conducted in the absence of any commercial or financial relationships that could be construed as a potential conflict of interest.

## Footnotes

1 Note that other proposals posit the origin of the “other-race” effect on the lifetime perceptual experience with those faces of the same race relative to those of the other race (e.g., Wan et al., 2015).

2 Studies exploring the encoding phase have focused mainly in the “Dm effect” (Differences based on later memory performance). Trials subsequently remembered (hits) and trials not remembered (misses) are compared.

3 The Cronbach alpha was also calculated for the time window between 300 and 500 ms. The results revealed that the internal reliability was moderate (values between 0.50 and 0.70) when 5 trials were considered (Hit-NL: 0.59; Hit-FL: 0.57; Crej: 0.51) and high when 10 trials were considered (Hit-NL: 0.79; Hit-FL: 0.82). For correct rejections, internal consistency was moderate for 10 trials (α = 0.61) and high when 15 trials were considered (α = 0.76). Altogether the reported Cronbach alpha values support the reliability of the reported old/new effects.

4 Average amplitudes for 13 trials was calculated for the 90% of participants (*n* = 30) in the NL and for 78% of participants in the FL (*n* = 25).

5 All these studies reported P200 effects in the recognition phase and not during the encoding one.
